# CD200-CD200R dysfunction exacerbates microglial activation and dopaminergic neurodegeneration in a rat model of Parkinson's disease

**DOI:** 10.1186/1742-2094-8-154

**Published:** 2011-11-06

**Authors:** Shi Zhang, Xi-Jin Wang, Li-Peng Tian, Jing Pan, Guo-Qiang Lu, Ying-Jie Zhang, Jian-Qing Ding, Sheng-Di Chen

**Affiliations:** 1 Department of Neurology & Institute of Neurology, Ruijin Hospital, Shanghai Jiao Tong University School of Medicine, 197 Ruijin Er Road, Shanghai 200025, P. R. China; 2 Laboratory of Neurodegenerative Diseases & key Laboratory of Stem Cell Biology, Institute of Health Science, Shanghai Institutes of Biological Sciences (SIBS), Chinese Academy of Science (CAS) & Shanghai Jiao Tong University School of medicine, 225 South Chong Qing Road, Shanghai 200025, P. R. China

## Abstract

**Background:**

Increasing evidence suggests that microglial activation may participate in the aetiology and pathogenesis of Parkinson's disease (PD). CD200-CD200R signalling has been shown to be critical for restraining microglial activation. We have previously shown that expression of CD200R in monocyte-derived macrophages, induced by various stimuli, is impaired in PD patients, implying an intrinsic abnormality of CD200-CD200R signalling in PD brain. Thus, further in vivo evidence is needed to elucidate the role of malfunction of CD200-CD200R signalling in the pathogenesis of PD.

**Methods:**

6-hydroxydopamine (6-OHDA)-lesioned rats were used as an animal model of PD. CD200R-blocking antibody (BAb) was injected into striatum to block the engagement of CD200 and CD200R. The animals were divided into three groups, which were treated with 6-OHDA/Veh (PBS), 6-OHDA/CAb (isotype control antibody) or 6-OHDA/BAb, respectively. Rotational tests and immunohistochemistry were employed to evaluate motor deficits and dopaminergic neurodegeneration in animals from each group. HPLC analysis was used to measure monoamine levels in striatum. Morphological analysis and quantification of CD11b- (or MHC II-) immunoreactive cells were performed to investigate microglial activation and possible neuroinflammation in the substantia nigra (SN). Finally, ELISA was employed to assay protein levels of proinflammatory cytokines.

**Results:**

Compared with 6-OHDA/CAb or 6-OHDA/Veh groups, rats treated with 6-OHDA/BAb showed a significant increase in counts of contralateral rotation and a significant decrease in TH-immunoreactive (TH-ir) neurons in SN. A marked decrease in monoamine levels was also detected in 6-OHDA/BAb-treated rats, in comparison to 6-OHDA/Veh-treated ones. Furthermore, remarkably increased activation of microglia as well as up-regulation of proinflammatory cytokines was found concomitant with dopaminergic neurodegeneration in 6-OHDA/BAb-treated rats.

**Conclusions:**

This study shows that deficits in the CD200-CD200R system exacerbate microglial activation and dopaminergic neurodegeneration in a 6-OHDA-induced rat model of PD. Our results suggest that dysfunction of CD200-CD200R signalling may be involved in the aetiopathogenesis of PD.

## Background

Parkinson's disease (PD) is the second most common neurodegenerative disease in the world, and is characterized by dopaminergic neuron loss in the substantia nigra pars compacta (SNpc) [1]. PD was first described by James Parkinson in 1817, and the aetiology of PD still remains unknown. However, emerging investigations suggest that multiple factors, both genetic and acquired, contribute to the loss of dopaminergic cells in the substantia nigra (SN) of these patients [2-4]. Among these culprits, accumulated evidence suggests that neuroinflammation, which is characterised by activation of microglia and subsequent production of proinflammatory cytokines, may play an important role in the neurodegenerative process in PD. Activated microglia are found in the SN of mesencephalon in the brain of PD patients [5-8] and of parkinsonian animal models [9-13]. Molecules related to neuroinflammation, such as tumor necrosis factor-alpha (TNF-α), IL-6, IL-1β, interferon-gamma (IFN-γ), and superoxide, have been found co-localized with microglia in brain, and in cerebrospinal fluid and serum of PD patients as well [6,7,14-22]. Taken together, those previous studies suggest that persistent activation of microglia is dynamically involved in the disease's progression.

CD200R, an important inhibitory receptor present on microglia [23], actively maintains microglia in a quiescent state through its interaction with CD200, a transmembrane glycoprotein expressed on neurons [24-29]. Recent publications have demonstrated that disruption of CD200-CD200R engagement can cause abnormal activation of microglia and consequent pathological changes. Microglia in CD200-deficient (CD200^-/-^) mice exhibit more characteristics of activation [30]. They are aggregated, less ramified and have shorter glial processes, as well as a disordered arrangement and increased expression of CD11b and CD45. Moreover, this increased microglial response is substantiated by enhanced expression of Class II major histocompatibility complex (MHC II), TNF-α and inducible nitric oxide synthetase (iNOS) [31]. Thus, CD200^-/- ^mice display earlier onset of experimental autoimmune encephalomyelitis (EAE) [30]. In addition, preventing CD200-CD200R interactions with CD200R-blocking antibodies also induces augmented microglial activation in EAE rats [32,33]. Conversely, CD200^-/- ^mice receiving exogenous CD200R agonist, including CD200 antigen [34] or an agonist anti-CD200R antibody [35], are resistant to the induction of experimental autoimmune uveoretinitis (EAU). All of these findings suggest that decreased interaction between CD200 and CD200R is related to increased activation of microglia. Interestingly, decreased expression of CD200 and CD200R have also been found in hippocampus and inferior temporal gyrus of patients suffering from Alzheimer's disease [36]. Down-regulation of CD200 has also been detected in brain of multiple sclerosis (MS) patients [37]. These results suggest that a deficient CD200-CD200R system may be involved in the progression of various neurological disorders [38,39]. Our previous study revealed altered regulation of CD200R in monocyte-derived macrophages from PD patients [40]. We also found that blocking CD200-CD200R engagement dramatically exacerbates dopaminergic neurodegeneration in a primary neuron/microglia co-culture system [41]. Thus, further in vivo evidence is needed to thoroughly elucidate the role of malfunction of CD200-CD200R signalling in the pathogenesis of PD. In the present study, we used a CD200R blocking antibody to destroy CD200-CD200R engagement in hemiparkinsonian rats, induced by 6-OHDA injection. We found that the impairment of CD200-CD200R interaction resulted in increased microglial activation and corresponding neurodegeneration in this animal model of PD.

## Methods

### Materials

Specific monoclonal antibodies against CD200R (CD200R-blocking antibody, BAb), CD11b, MHC II and isotype control mouse IgG1 (Control antibody, CAb) were obtained from Serotec (Indianapolis, IN, USA). The ELISA kit for rat-TNFα was obtained from R&D Systems (Minneapolis, MN, USA). The ELISA kit for rat-IL-6 was purchased from BD (San Diego, CA, USA). Elite ABC kit and 3,3'-diaminobenzidine tetra-hydrochloride (DAB) substrate were purchased from Vector (Vector Laboratories, Burlingame, CA, USA). The BCA Protein Assay Kit was from Thermo Fisher Scientific (Rockford, IL, USA). High-performance liquid chromatography (HPLC)-grade methanol was obtained from BDH Laboratory (Poole, UK). All other chemicals were obtained from Sigma-Aldrich (St. Louis, MO, USA).

### Animals

All animal experiments were performed according to the NIH Guide for the Care and Use of Laboratory Animals and were approved by the Shanghai Jiao Tong University School of Medicine Animal Care and Use Committee (2009087). Male Sprague-Dawley rats (10-12 weeks old, weighing 220-260 g at the start of the experiment) were provided by the Shanghai Institutes of Biological Sciences animal house, and were caged in groups of 5 with food and water given ad libitum. The animals were kept in a temperature-controlled environment at 22 ± 2°C on a 12:12 light-dark cycle.

### Steoreotaxic surgery

For stereotaxic surgery, rats were anesthetized with an intraperitoneal injection of pentobarbital (50 mg/kg). When the animals were deeply anesthetized, they were placed in a stereotactic apparatus. Subsequently, the rats were injected with BAb (1 μg/μl, 5 ul for each site) or CAb (1 μg/μl, 5 ul for each site) into the right striatum (anterior lesion site: AP: 1.0 mm anterior to the bregma, L: 2.6 mm from the midline, D: 4.5 mm from the dura; posterior lesion site: AP: 0.3 mm posterior to the bregma, L: 3.5 mm from the midline, D: 4.5 mm from the dura). The sham groups were injected with vehicle (10 mM PBS, 5 μl for each site, Veh). The next day, each group was injected with 6-OHDA (4 μg/μl in 0.9% saline with 0.02% ascorbic acid, 2 μl for each site) into the right ascending medial forebrain bundle (MFB) (one 4.2 mm posterior to bregma, 1.2 mm lateral to the midline, and 7.8 mm below the dura, and another 4.4 mm posterior to bregma, 1.7 mm lateral to the midline, and 7.8 mm below the dura). The microinjection coordinates used were obtained from a rat brain atlas by Paxinos and Watson. The injection was made at a rate of 1 μl/min using a 10 μl Hamilton syringe with a 26-gauge needle. At the end of each injection, the syringe needle was left in place for 5 min, and then was slowly withdrawn to prevent reflux of the solution.

### Tissue preparation

At 21 days post 6-OHDA-injection, animals were deeply anesthetized with pentobarbital (100 mg/kg, i.p.) and perfused through the aorta with 150 ml of 0.9% saline, followed by 250 ml of a cold fixative consisting 4% paraformaldehyde in 100 mM phosphate buffer (PB). Brains were then dissected out (3-4 mm in thickness) and postfixed for 24 hours with paraformaldehyde in 100 mM PB before placed into 30% sucrose solution in phosphate-buffered saline for 24-72 hours at 4°C. Brains were then cryosectioned coronally on a Leica1650 cryostat (cut thickness: 25 μm) with a random start, and including sections before and after both anatomical regions to confirm the entire structure was quantified. Sections were collected serially throughout the SN and placed into PBS for further experiments.

### Immunohistochemistry

Free-floating sections were pretreated with 0.3% H_2_O_2 _in 0.1 M PBS (pH 7.2-7.5) for 10 min at RT (60 rpm) to block endogenous peroxidase activity, then washed with 0.1 M PBS for 3 times. The tissue was then blocked with diluted blocking serum (Elite ABC kit, Vector Laboratories, Burlingame, CA, USA) for 20 minutes at room temperature. Sections were then incubated with the primary antibody to TH (mouse anti-TH, 1:4000, Sigma), CD11b (mouse anti-CD11b, 1:1000, serotec) or MHC II (mouse anti-MHC II, 1:1000, serotec) overnight at 4°C. The following day the sections were washed and then incubated with diluted biotinylated secondary antibody (Vector laboratories) for 30 min at room temperature. The secondary antibody was amplified using avidin-biotin complex (Vector laboratories) for 30 min at room temperature. Finally the sections were developed with 3,3'-diaminobenzidine tetra-hydrochloride (Vector Laboratories). Sections were then mounted onto glass slides and dried overnight. The next day the slides were passed through a gradient of ethyl alcohol and xylene to dehydrate the tissue. The slides were then coverslipped using permount mounting medium.

### Cell quantification

Unbiased stereological estimates of DA (TH-positive cell) neuron numbers were performed using StereoInvestigator analysis software (MicroBrightField, Williston, VT), combined with a Nikon Eclipse E600 microscope, and the optical fractionator method according to previously published reports [42,43]. Boundaries in the SN were defined according to previously defined anatomical analysis in the rat [44] and cells were counted from every sixth 25-μm section (~24 sections) along the entire SN (to ensure coefficient of errors <0.1, the rostral-caudal length of the SN was 4 mm), by investigators blinded to treatment history, with a 60 × objective. In brief, optical dissectors (area of counting frame, 64,000 mm^3^; guard height, 2 μm; spaced 300 μm apart in the x-direction, and 200 μm apart in the y-direction) were applied to each section in the series throughout the entire SN (including pars reticulata and compacta; estimates are reflective of two sides; n = 5 for each group). We show the percent of neurons remaining on the ipsilateral side compared to those on the intact contralateral side. Values are expressed as the mean ± S.E.M. of all animals in each group.

Microglial quantification similarly used adjacent (8 sections) serial sections. An observer blind to sample identity counted numbers of CD11b-immunoreactive (CD11b-ir) positive cells in the SN on each side (Nikon microscope at a 40 × magnification). Here the X-Y step length used was between 300-400 mm in order to count 100-200 CD11b-ir cells in each side of the SN. A positive cell was defined as a nucleus covered and surrounded by CD11b immunostaining. The stage of cells was identified by their morphology. For quantitation of MHC II immunoreactive (MHC II-ir) cells, cells in stage 4 were identified by their morphology on MHC II staining under 40× magnification and counted in every sixth 25-μm-thick serial section of the SN of each rat using a two-blinded procedure. Graphs show the number of MHC II-ir cells in the SN.

### Measurements of dopamine and its metabolites by HPLC

Animals (n = 5 each of the following groups: 6-OHDA/Veh, 6-OHDA/CAb, and 6-OHDA/BAb) were sacrificed by CO_2 _and their brains were quickly removed and placed on ice. The left and right striatum were freshly dissected out, weighed, frozen in liquid nitrogen, and stored at -80ºC for later use.

Each sample was homogenized by sonication in ice-cold 0.1 mol/L perchloric acid and then centrifuged at 12,000 rpm for 30 minutes at 4ºC. The supernatants (20 μl) were injected into a high-performance liquid chromatography (HPLC) system coupled to an electrochemical detection device (Coularray; ESA, Chelmsford, MA) for measuring dopamine (DA), 3,4-dihydroxyphenylacetic acid (DOPAC) and homovanillic acid (HVA). The protein contents were determined in pellet fractions by the method described by Lowry [45], and expressed as ng/g wet weight of tissue (ng/g WW).

### Classification of microglial activation

We adapted a classification system for microglial activation according to Kreutzberg [46]:

Stage 1: Resting microglia. Rod-shaped soma with fine and ramified processes.

Stage 2: Activated ramified microglia. Elongated cell body with long and thicker processes.

Stage 3: Amoeboid microglia. Round body with short, thick and stout processes.

Stage 4: Phagocytic cells. Round cells with vacuolated cytoplasm; no processes can be observed at the light microscopy level.

Stages of microglia activation were confirmed by observation by at least two blinded observers. Black circles in Figure 2 show examples of microglia in different stages. All of these cell types are CD11b-ir, and MHC II stained only activated microglia but not resting microglia.

### Rotational behaviour

Apomorphine-induced rotational behaviour was assessed at 7 and 21 days after 6-OHDA-injection. Rotational behaviour was tested in rotometer bowls [47]. Five minutes after intraperitoneal administration of apomorphine (0.5 mg/kg diluted in 0.9% saline), the total number of full 360° rotations in the contralateral direction was counted for 30 min.

### ELISA for TNF-α and IL-6

Rats were killed by CO_2 _overdose followed by cervical dislocation and decapitation at 21 days after injection of 6-OHDA. The brain was removed and immediately transferred to ice and cut at the level of the infundibular stem forming a hindbrain block containing the SN. The SN were dissected, snap-frozen in liquid nitrogen and stored at -80°C. Tissue was homogenized on ice in 400 μl of Tris-HCl buffer (pH = 7.3) containing protease inhibitors (10 mg/ml aprotinin, 5 mg/ml peptastin, 5 mg/ml leupeptin, 1 mM PMSF). Homogenates were centrifuged at 10,000 g at 4ºC for 10 min and then ultracentrifuged at 40,000 r.p.m. for 2 h. Supernatants were aliquoted and stored at -80ºC until use. BCA protein assays were performed to determine total protein concentration in each sample. Commercially available rat TNF-α (R&D, Minneapolis, MN, USA and rat IL-6 kits (BD, San Diego, CA, USA) with high sensitivity were used to quantify these cytokines according to the manufacturers' instructions (7.8 pg/ml for rTNF-α and 20 pg/ml for rIL-6). Three animals per group were analyzed and each sample was analyzed in duplicate.

### Statistical analysis

Statistical analysis of the data was performed using GraphPad Prism version 5.00 for Windows (GraphPad Software, San Diego California, USA, http://www.graphpad.com). The results are reported as mean ± SEM. Two-way ANOVA followed by Bonferroni's test was applied to determine significant differences among data of rotational experiments with two time points. Univariate one-way ANOVA and Tukey-Kramer post-hoc test were used to analyze data from other experiments between treated group**s**. The criterion for statistical significance was P < 0.05.

## Results

### BAb administration enhances rotational asymmetry in 6-OHDA-induced hemiparkinsonian rats

**Figure 1:**
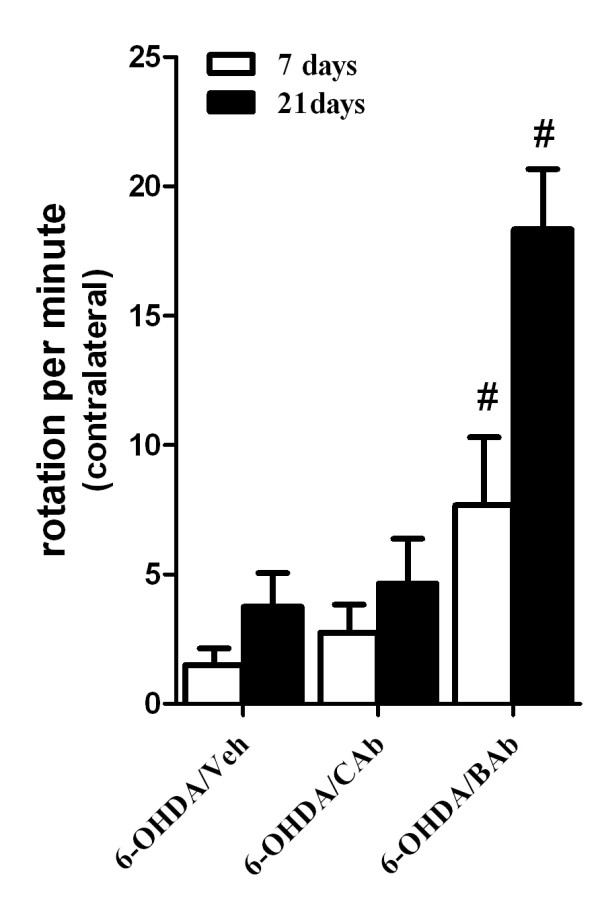
**BAb exacerbates 6-OHDA-induced behavioural deficits**. Contralateral rotation measurements following administration of apomorphine in each experimental group are shown in bar graph at 7 days and 21 days post-6-OHDA injection. Data are presented as mean ±S.E.M (n = 5 rats/group). (#)Statistical differences from 6-OHDA/Veh- or 6-OHDA/CAb-treated animals are P < 0.001.

Unilateral injection of 6-OHDA into medial forebrain bundle (MFB) induces the loss of dopaminergic cell in the ipsilateral SNpc and was used as a hemiparkinsonian animal model in this study. To investigate the role of CD200-CD200R dysfunction in 6-OHDA-induced neurotoxicity, we employed a CD200R monoclonal antibody to block CD200-CD200R engagement, which was first used by Wright, G. J. [32], and later by many other investigators [33,41,48-51]. In the present study, BAb, CAb or Veh was injected into striatum one day before 6-OHDA injection. Then, apomorphine-induced rotational behaviour was analyzed to assess unilateral degeneration of presynaptic dopaminergic neuron terminals at 7 and 21 days after 6-OHDA injection. Although rats that had been microinjected with 6-OHDA/Veh could contralaterally rotate to the site of 6-OHDA lesion with apomorphine administration (1.5 ± 0.6 at 7 days, 3.7 ± 1.3 at 21 days), apomorphine-induced rotation was significantly increased in 6-OHDA/BAb rats at both time points (7.7 ± 2.6 and 18.3 ± 2.3 respectively, p < 0.0001) (Figure 1). Pretreatment with BAb not only exacerbated but also accelerated (as early as 7 days) motor deficits in hemiparkinsonian rats (Figure 1). Animals that responded to apomorphine treatment with at least 7.0 turns/min could be regarded as successfully induced hemiparkinsonian rats [43,52]. The rats that received 6-OHDA/Veh treatment showed only 1.5 ±0.6 turns/min at 7 days and 3.7 ±1.3 turns/min at 21 days induced by apomorphine administration, and both of these values are less than 7.0 turns/min. So the dose of 6-OHDA (16 μg) used in this experiment could be considered as a sub-toxic dose. However, rats treated with 6-OHDA/CAb did not show a significant increase in contralateral rotational number, 2.7 ± 1.1 turns/min at 7 days and 4.7 ±1.7 turns/min at 21 days, compared to the rats treated with 6-OHDA/Veh (Figure 1).

### BAb administration exacerbates 6-OHDA-induced neurodegeneration

**Figure 2:**
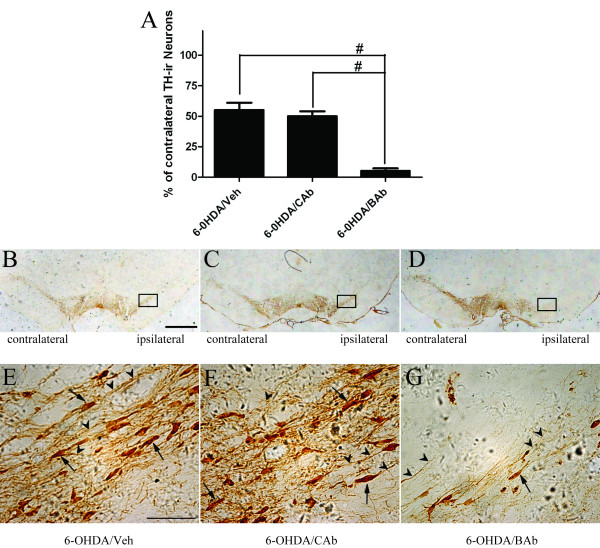
**Neurodegeneration is exacerbated in the SN of hemi-parkinsonian rats**. Animals (5 rats per groups) were sacrificed at 21-day post 6-OHDA injection to characterize and quantify loss of dopaminergic neurons in whole SN. (B-G) Representative sections (~24 sections per rat) of SN were immunostained with antibodies against tyrosine hydroxylase (TH). (B,E) SN of 6-OHDA/Veh group, (C,F) SN of 6-OHDA/CAb group, (D,G) SN of 6-OHDA/BAb group. The bottom photos (E-G) are higher magnifications of the cells in black rectangles of ipsilateral SN in the upper photos (B-D). Scale bar: 1 mm (B-D); scale bar: 50 μm (E-G). Arrows: neurons; arrowheads: fibers. (A) Stereological cell counts of total TH-ir neurons on the ipsilateral side of the SN are shown as a percentage of the cells on the contralateral side (n = 5/group, # P < 0.0001). All the results were obtained in a two-blinded procedure. No significant difference was found between the group treated with 6-OHDA/Veh and the group treated with 6-OHDA/CAb (P = 0.5108). Data are presented as mean ± S.E.M.

To confirm that the phenotype of our PD rats is consistent with dopaminergic neuron loss in SN, we stained midbrain coronal sections with an antibody against tyrosine hydroxylase (TH) and performed non-biased stereological estimation of TH-immunoreactive (TH-ir) neurons in SN. We observed that a sub-toxic dose of 6-OHDA was able to induce moderate but not overt dopaminergic neurodegeneration in SN (55.0 ± 6.0% of contralateral) (Figure 2A). Intrastriatal injection of BAb resulted in a significant decrease in TH-ir neurons in whole SN in animals treated with 6-OHDA/BAb (5.2 ±2.0%, P < 0.0001). However, no dramatic decrease in TH-ir cells was observed between groups treated with 6-OHDA/Veh and 6-OHDA/CAb (Figure 2A). These results indicate an exacerbating effect of BAb on the degeneration of dopaminergic neurons. At higher magnifications, we observed that treatment of 6-OHDA/BAb not only decreased the number of TH-ir cells but also their arborisation or fibers. TH-ir fibers (Figure 2 arrowheads) were less densely spread amongst TH-ir cell bodies (Figure 2 arrows) in the SN in 6-OHDA/BAb-treated rats (Figure 2G), compared to either control group (6-OHDA/Veh, 6-OHDA/CAb) (Figure 2E-F). There were no marked morphological differences in numbers of TH-ir cells in SN between rats pretreated with CAb (6-OHDA/CAb) and Veh (6-OHDA/Veh) (Figure 2E-F). Furthermore, BAb administration had a significant effect on DA and its metabolites in ipsilateral striatum. Twenty-one days after 6-OHDA injection, the DA content of right striatum in 6-OHDA/Veh-treated rats was 1765 ± 236 ng/g wet weight of tissue (ng/g WW) (n = 5) (Table 1). Protein levels of DA metabolites in this group were 894 ± 95 ng/g WW (n = 5) and 599 ± 104 ng/g WW (n = 5) for DOPAC and HVA respectively (Table 1). Injection of CAb prior to 6-OHDA-lesion did not cause significant changes in DA or its metabolites in the right striatum of rats in comparison to vehicle control animals (Table 1), while the contents of DA and its metabolites in 6-OHDA/BAb group were significantly lower than that in the 6-OHDA/Veh group. These values were 38 ± 8 ng/g WW (n = 5), 24 ±3 ng/g WW(n = 5) and 17 ±2 ng/g WW (n = 5), respectively (Table 1).

**Table 1:** BAb administration increases 6-OHDA-induced dopamine deficiency in ipsilateral striatum.

Groups	Content (ng/g Wet weight of tissue)		
	
	DA	DOPAC	HVA
6-OHDA/Veh	1765.0 ± 235.6	894.1 ± 94.7	598.8 ± 103.6

6-OHDA/CAb	1674.0 ± 174.9	836.7 ± 72.3	551.7 ± 121.5

6-OHDA/BAb	37.6 ± 7.8 ***	24.2 ± 3.2 ***	16.7 ± 2.3 **

**p < 0.01, ***p < 0.001 compared to the 6-OHDA/Veh and 6-OHDA/CAb groups (n = 5 per group)			

Results are expressed as mean ± S.E.M. (ng/g WW) of total protein. Data are shown only for ipsilateral (right) side of striatum. Statistical analysis was performed by two-way ANOVA. ** p < 0.01, *** p < 0.001 as compared to 6-OHDA/Veh lesion group.

### BAb treatment exacerbates 6-OHDA-induced microglial activation

The direct effect of BAb is destruction of the balance between CD200 and its receptor. CD200R receptor is expressed only on microglia [24,53,54].

Signal transferred from CD200 to its only known receptor, CD200R, has been shown to be critical for restraining microglial activation [30]. Thus, we studied microglial activation and possible neuroinflammation in SN at 21 days post-6-OHDA-injection by immunohistochemistry as described in Methods.

**Figure 3:**
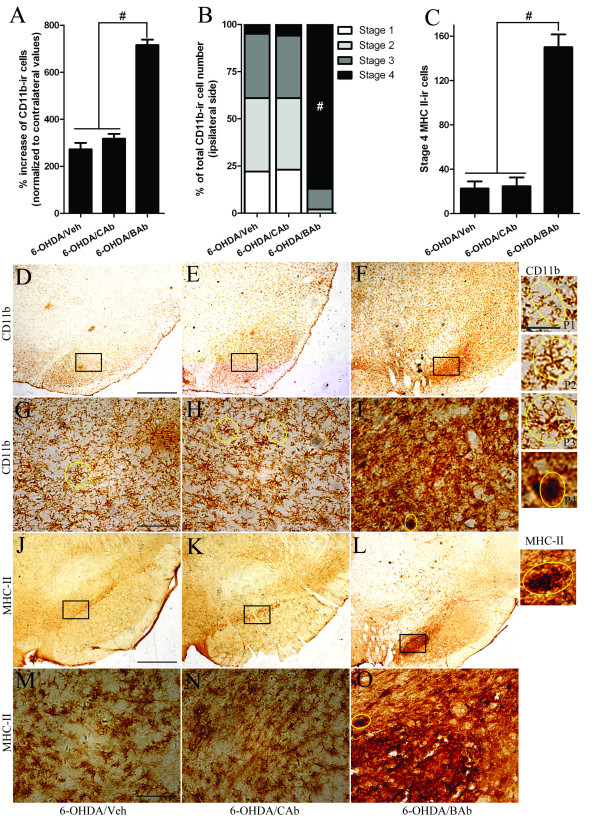
**Effects of BAb on microglial morphology and cell number in SN**. Representative sections of SN in different groups were immunostained with antibodies against CD11b (a microglia marker) (D-I) and MHC II (a marker for activated microglia) (J-O) 21 days after 6-OHDA-injection. G-I and M-O are higher magnifications of the fields outlined by rectangles in D-F and J-L respectively. Scale bar: 500 μm in D-F and J-L; scale bar: 50 μm in G-I and M-O. Representative microglia in different stages (stage1-4) of CD11b immunostaining are shown in yellow circles (stage1: P1; stage2: P2; stage3: P3; stage4: P4), and a representative of stage 4 MHC II-ir microglia is shown in panel Q. Scale bar:10 μm in P1-P4 and Q. Microglia cell numbers and morphology were stereologically analyzed in each group. (A) Data represents average increase of CD11b-ir microglia cell number in ipsilateral SN as compared to contralateral SN (n = 5) ± S.E.M. #:P < 0.001. (B) Stereological quantification of each stage of CD11b-ir microglia is depicted as the average percentage distribution per group. # p < 0.001 compared to every other group. (C) Stereological quantification of stage 4 MHC II-ir cells throughout the SN from different experimental groups is shown in bar graph; n = 5, value = mean ± S.E.M. #: P < 0.001 significant difference compared to every other group.

**Figure 4:**
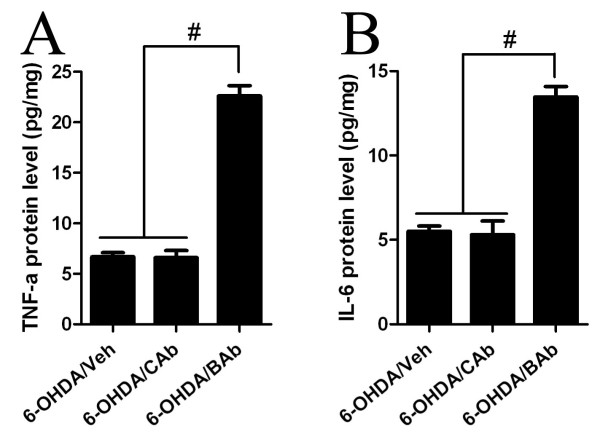
**BAb regulates pro-inflammatory factor production in SN**. Concentrations of TNF-α and IL-6 in SN were assayed using ELISA at 21 days post-6-OHDA injection. Values are shown as mean ± S.E.M. The concentrations of cytokines in the 6-OHDA/BAb co-treated group were significantly higher than those in the other groups. There was no significant difference between the 6-OHDA/CAb- and the 6-OHDA/Veh-treated groups. Data are representative of three individual experiments. # P < 0.001, significant difference compared to every other group.

First, we studied morphological changes and quantification of microglia using the microglia-specific marker CD11b (a constitutive marker of microglia). CD11b recognizes complement receptor type 3 (CR3), the expression of which is greatly increased in hyperactive microglia compared with resting microglia. In our study, profound microglial responses were observed in ipsilateral SN in rats following treatment with 6-OHDA/BAb (Figure 3F,I). Round and amoeboid cells (Stage 4) became predominant in the core of the SN and were mingled with rod-shaped (stage 3) or highly ramified (stage 2) microglia near the boundary (Figure 3I). Twenty one days post-6-OHDA injection there was an increase in CD11b-ir cells in all groups. In addition, we found that the total number of CD11b-ir cells in SN was significantly increased in 6-OHDA/BAb-treated rats (716 ± 23%, P = 0.0002) versus 6-OHDA/CAb-treated rats (318 ± 20%) and 6-OHDA/Veh-treated rats (273 ± 27%) (Figure 3A). No significant difference was found between 6-OHDA/Veh and 6-OHDA/CAb groups (P = 0.2519) (Figure 3A). Furthermore, we analysed the quantification of microglia in different stages. Four cellular patterns (Figure 1, 2, 3, 4) were defined according to Kreutzberg's classification [46]. We observed that stage 4 cells constituted over 85% of the microglia population in the 6-OHDA/BAb-treated group, while less than 10% of them were found in the other two groups (Figure 3B). The majority of cells presenting in the core lesion of the SN in the other two groups were stage 3 cells or stage 2 cells. In the 6-OHDA/Veh group, stage 3 cells constituted about 34% of the population, while 39% of population turned out to be stage 2 cells. In the 6-OHDA/CAb group, 33% of population were stage 3 cells, while 38% were stage 2 cells. Statistically significant differences were found between the 6-OHDA/BAb-treated group and the two control groups (P < 0.01), but no difference was found between the two control groups. We also investigated the expression of MHC II, a marker for activated microglia, which is practically undetectable in resting microglia. MHC II-ir cells showed similar morphology to that of stage 4 microglia. MHC II-ir microglia were found scattered throughout the SN in the 6-OHDA/Veh and 6-OHDA/CAb groups (Figure 3M-N), while mainly stage 4 MHC II-ir microglia were visualized in the core lesion of SN from the 6-OHDA/BAb treated group (Figure 3L,O). The number of stage 4 MHC II-ir microglia was dramatically increased in 6-OHDA/BAb-treated rats compared with the two control groups (n = 5, P < 0.001) (Figure 3C). No difference was detected between the two control groups.

Taken together, these data suggest that BAb administration shifts stage 2 or stage 3 microglia to stage 4 in SN. These results also show a distinct population of activated microglia (MHC II-ir), which correlates with levels of neurodegeneration and motor deficit in the 6-OHDA/BAb group.

### BAb treatment increases 6-OHDA-induced proinflammatory factors production in SN

To further confirm a relationship between CD200-CD200R signalling and neuroinflammation in PD, we assayed several molecules that would be secreted by activated microglia in the proinflammatory stage [10,55-59]. We detected the expression profile of two most-important cytokines, TNF-α and IL-6, in SN of rats from each group at 21 days post-6-OHDA-injection. This is the time point at which dopaminergic neurodegeneration, rotational behaviour and microglial activation were investigated. Interestingly, we found significant increases in the induction of TNF-α and IL-6 expression in rats treated with 6-OHDA/BAb in comparison with the other treatments (6-OHDA/CAb, 6-OHDA/Veh) (Figure 4, P < 0.001). No difference was noted between the two control groups (Figure 4). Thus we speculate that these two cytokines, TNF-α and IL-6, might be involved in the exacerbating effects observed in the 6-OHDA/BAb-treated animals.

## Discussion

We sought in vivo evidence for a role for CD200-CD200R dysfunction in the etiopathogenesis of PD. Microglia, which are not only the resident innate immune cells in the CNS [23,46] but also the predominant cells that express CD200R in CNS [60], play a critical role in maintaining a homeostatic milieu for most vulnerable dopaminergic neurons. CD200-CD200R signalling is considered to be a brake on innate immunity [61]. Breaking the interaction between CD200 and CD200R will cause abnormal activation of microglia in brain.

Normal CD200-CD200R signalling maintains microglia in a quiescent state. Hoek et al. [30] first reported that disruption of CD200-CD200R interaction in the nervous system can cause EAE, which is related to abnormal activation of microglia. Recently, several studies have shown links between CD200/CD200R signalling and PD, Alzheimer's disease (AD) and prion diseases. Protein and mRNA levels of CD200 and CD200R are decreased in hippocampus and inferior temporal gyrus of AD patients [36], suggesting that deficiency of the CD200-CD200R signalling may play an important role in the progress of AD [36]. Costello et al. [62] observed an exaggeration of proinflammatory cytokine production, including IL-1β, IL-6 and TNF-α, produced by CD200^-/- ^glia And these up-regulated cytokines correlated with significantly reduced long-term potentiation (LTP) at CA1 synapses of hippocampal slices from CD200^-/- ^mice [62]. These findings indicated that loss of CD200-CD200R interaction might impair synaptic function in hippocampus and play an important role in dementia. A deficit of CD200-CD200R has also been found in PD patients. Luo et al. [40] examined CD200R expression and regulation in monocyte-derived macrophages (MDMs), the peripheral counterpart of microglia, in PD patients and in old and young healthy controls. They found that basal CD200R expression is similar in MDMs from young control, old control and PD patients; however, expression of CD200R in MDMs induced by various stimuli is impaired in the older groups, especially in PD patients, implying an intrinsic abnormality of CD200-CD200R signalling in PD brain. Interestingly, CD200R expressed in human beings and rats functions only as an inhibitory signal [60]. However there are two different CD200Rs in mice [54,60,63,64]; an inhibitory receptor CD200R1 [48,65-68] and an activating receptor CD200R2-4 [69]. There is no report about the expression levels of CD200R or CD200 in patients with prion disease, but activated microglia are thought to be related to up-regulation of CD200R4 in a mouse model of prion disease [70]. All of these findings suggest that CD200-CD200R signalling plays an important role in the pathogenesis of neurological disorders, including PD.

Previously, we always used 32μg of 6-OHDA to yield an animal model of PD [43,52]. This amount would result in the demise of almost all dopaminergic neurons in the SN (>95%) and in the ventral tegmental area (VTA) (>80%) at 3 weeks post-lesion [43,52]. To investigate whether abnormal CD200-CD200R signalling could exacerbate microglial activation and dopaminergic neurodegneration in the 6-OHDA-induced rat PD model, we needed to find a proper dose of 6-OHDA that would produce only a limited loss of TH-ir neurons on the ipsilateral side of the SN. Therefore, we injected different amounts (32μg, 24μg, 16μg, 8μg) of 6-OHDA into MFB and found that 16μg of 6-OHDA was able to induce moderate but not overt dopaminergic neurodegeneration in SN (data not shown). This is the sub-toxic dose of 6-OHDA that is similar to that used by Saucer H et al. [71], Depino AM et al. [12] and Roedter A et al. [72]. In these studies, 20μg 6-OHDA in the striatum provoked a moderate and progressive loss of dopaminergic cells in the ipsilateral SN at 3 weeks post -lesion. The typical phenotype and corresponding neurodegeneration, as well as augmented microglial activation, observed in 6-OHDA/BAb-treated rats suggests that abnormal CD200-CD200R signalling exacerbates microglial activation and plays an important role in progression of the disease. It is believed that multiple factors are involved in the development of PD. Our present study in a PD rat model and our previous study in PD patients indicate that both intrinsic abnormal CD200-CD200R signalling and environmental neurotoxins participate in the pathogenesis of PD.

According to previous studies, the bolus administration of any substance into cerebrum may cause mechanical damage to neurons [73,74] and subsequent adjacent activation of microglia [74-79]. This makes it difficult to distinguish activation of microglia caused by injection from that caused by changes in CD200-CD200R signalling. Beside this, the small volume of the SN makes it hard to inject any reagent precisely into the SN [80,81]. Finding an ideal alternative antibody injection site would help to elucidate the role of CD200-CD200R signalling in the pathogenesis of PD. Phaseolus vulgaris-leucoagglutinin and biocytin, injected into striatum, can later be found in substantia nigra pars reticulate (SNpr) and substantia nigra pars compacta (SNpc) in squirrel monkeys [82]. In addition, Mufson et al [83] have shown that intrastriatral infusion of the tracer fluorogold results in transport into the SNpc. The above evidence indicates that antibody injected into striatum may spread into the SN, causing abnormal activation of microglia and damage to dopaminergic neurons. Histological and immunological examinations in rats confirmed our speculation. Furthermore, the reduced levels of DA and its metabolites caused by injection of BAb in striatum demonstrates impairment of dopaminergic neurons in SN.

The results of this study provide in vivo evidence that impairment of CD200-CD200R signalling might play an important role in the pathogenesis of PD. However, our study lacked a time course of microglial activation and neuroinflammation. Therefore, further study is required to fully elucidate the mechanism involved in microglial activation and subsequent neurodegeneration.

## Conclusions

Taking all of these results together, this study shows that disruption of CD200-CD200R signalling might play a role in the pathogenesis of PD. The role of CD200-CD200R signalling in the pathogenesis of PD makes it a potential therapeutic target for PD therapy. Therapeutic agents that can efficiently inhibit microglial activation through regulation of CD200-CD200R signalling may become a novel approach to the clinical treatment of PD.

## Competing interests

The authors declare that they have no competing interests.

## Authors' contributions

SZ, XJW, JQD, SDC designed research. SZ, LPT, JP, GQL, YJZ performed research. SZ wrote paper. All authors read and approved the final manuscript.
